# Badger macrophages fail to produce nitric oxide, a key anti-mycobacterial effector molecule

**DOI:** 10.1038/srep45470

**Published:** 2017-04-06

**Authors:** Kirstin Bilham, Amy C. Boyd, Stephen G. Preston, Christina D. Buesching, Chris Newman, David W. Macdonald, Adrian L. Smith

**Affiliations:** 1 Department of Zoology, University of Oxford, South Parks Road, OX1 3PS, United Kingdom; 2 Wildlife Conservation Research Unit Department of Zoology, University of Oxford, The Recanati-Kaplan Centre, Tubney House, Abingdon Road, Tubney, Abingdon OX13 5QL, United Kingdom

## Abstract

The European badger is recognised as a wildlife reservoir for bovine tuberculosis (bTB); the control of which is complex, costly and controversial. Despite the importance of badgers in bTB and the well-documented role for macrophages as anti-mycobacterial effector cells, badger macrophage (bdMφ) responses remain uncharacterised. Here, we demonstrate that bdMφ fail to produce nitric oxide (NO) or upregulate inducible nitric oxide synthase (iNOS) mRNA following Toll-like receptor (TLR) agonist treatment. BdMφ also failed to make NO after stimulation with recombinant badger interferon gamma (bdIFNγ) or a combination of bdIFNγ and lipopolysaccharide. Exposure of bdMφ to TLR agonists and/or bdIFNγ resulted in upregulated cytokine (IL1β, IL6, IL12 and TNFα) mRNA levels indicating that these critical pathways were otherwise intact. Although stimulation with most TLR agonists resulted in strong cytokine mRNA responses, weaker responses were evident after exposure to TLR9 agonists, potentially due to very low expression of TLR9 in bdMφ. Both NO and TLR9 are important elements of innate immunity to mycobacteria, and these features of bdMφ biology would impair their capacity to resist bTB infection. These findings have significant implications for the development of bTB management strategies, and support the use of vaccination to reduce bTB infection in badgers.

European badgers (*Meles meles*) are implicated as a major wildlife reservoir for bovine tuberculosis (bTB), the control of which poses substantial practical and political challenges^1^,^2^,^3^. In the United Kingdom (UK), attempts to control the spread of bTB cost the taxpayer c. £99 m/annum (2013–14)^4^, yet the problem continues to worsen and control measures have sparked great controversy^5^. Badger culling has been implemented in bTB endemic areas despite strong protests^6^ yet the efficacy of culling strategies has been questioned^7^,^8^. Vaccination of badgers with Bacillus Calmette–Guérin (BCG) is efficacious under laboratory and field conditions^9^,^10^ and a current initiative is examining how badger vaccination can control bTB^11^. Understanding of badger immune responses to bTB challenge is limited despite the potential for this research to better inform bTB control efforts as well as enhancing vaccine development and deployment strategies. Here we present a critical study of badger innate immunity focussing on macrophages, TLR-based pathogen recognition and outcomes of macrophage activation.

Macrophages are the principal target cell for the growth of mycobacteria and key effector cells in immunity. Efficient anti-mycobacterial responses require induction of strong nitric oxide (NO) responses and mice deficient in inducible nitric oxide synthase (iNOS) are highly susceptible to TB^12^. Macrophages produce NO by upregulation of the iNOS gene in response to stimulation by Toll-like receptor (TLR) agonists and/or cytokines such as interferon gamma (IFNγ)^13^. Mammals typically express 10–12 TLRs that recognise different pathogen-associated molecular patterns (PAMPs)^14^ and initiate a signalling cascade, triggering macrophages to produce cytokines (including IL1β, IL6, IL12 and TNFα) and enter an enhanced antimicrobial state (reviewed in ref. 15). In humans, single nucleotide polymorphisms in TLR1, 2 and 9 associate with increased susceptibility to TB^16^,^17^ and in murine models, TLR2 and TLR9 knockouts exhibit increased levels of TB replication^18^. We investigated NO production and cytokine mRNA upregulation in bdMφ following activation of TLR and IFNγ pathways revealing two aspects of macrophage function in badgers relevant to bTB susceptibility and vaccination strategies.

## Results

### Badger macrophages do not produce nitric oxide and fail to effectively upregulate iNOS gene expression

**Figure 1:**
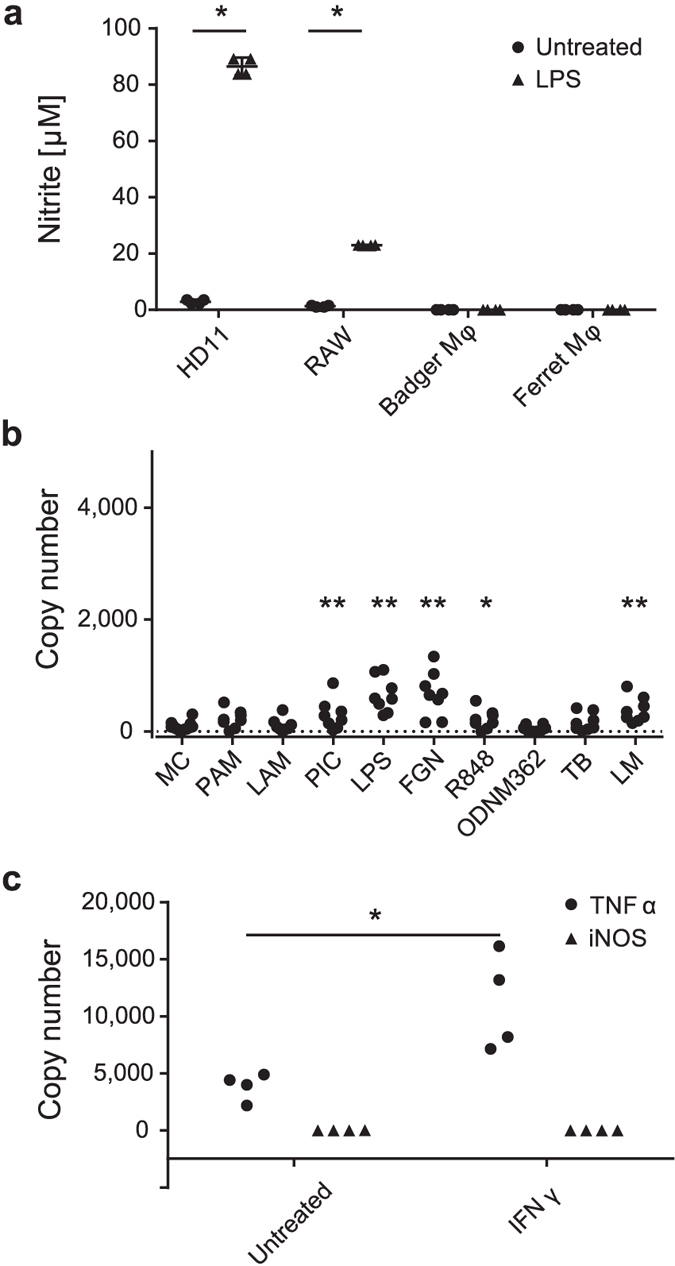
Badger macrophages do not produce NO. (**a**) Badger and ferret peripheral blood monocyte-derived macrophages, and mouse and chicken macrophage cell lines, were treated with LPS and supernatants assayed for NO after 48 hours (MC: media control). Error bars indicate standard error of the mean. (**b**) QRT-PCR was used to measure iNOS RNA in bdMφ following TLR agonist treatment. GAPDH was detected at a mean of 1.1 × 10^6^ copies/well. (**c**) Badger macrophages were treated with recombinant badger IFNγ and induction of TNFα and iNOS measured by QRT-PCR. QRT-PCR results are given as copy number/well. GAPDH = 1.5 × 10^5^ copies/well. Difference from media control (MC): *p < 0.05, **p < 0.01.

Forty-eight hour cultured badger blood monocyte-derived adherent cells exhibited classical macrophage morphology and were phagocytic (Supplementary Fig. 1). Badger macrophages did not produce NO detectable by Griess assay following 48 hours exposure to lipopolysaccharide (LPS, Fig. 1a). In contrast, similar treatment of murine and chicken macrophages resulted in substantial NO production (Fig. 1a). Despite extensive analyses with macrophages isolated from wild badgers (n = 33) NO was never detected in response to a wide range of TLR agonists (Pam3CSK4, PAM; lipoarabinomannan, LAM; poly (I:C), PIC; flagellin, FGN; R848; ODN M362) or heat killed TB, BCG or *Listeria monocytogenes* (LM). To test whether this lack of NO phenotype was badger-specific or found in another Mustelid, we examined macrophages derived from ferret peripheral blood monocytes (Fig. 1a), or spleen and neither produced detectable NO after exposure to LPS, or other TLR agonists.

The complete absence of NO may have been due to a disruption in the badger iNOS gene. To test for this possibility, we designed primers targeting conserved regions (using human, mouse, dog, ferret and giant panda iNOS genome sequence). A putative badger iNOS gene fragment was obtained by PCR, cloned and the sequence verified. This sequence was used to develop a QRTPCR, which revealed that the iNOS mRNA signal was very low in untreated and TLR agonist-treated Mφ or those treated with heat-killed bacterial preparations (<1000 copies/1.1 million copies of GAPDH) (Fig. 1b). In comparison, TLR agonist treated murine Mφ upregulate iNOS mRNA to levels equivalent to the levels of GAPDH signal^19^. Hence, the very low iNOS mRNA levels detected with bdMφ are consistent with the total lack of NO response.

IFNγ initiates a TLR-independent pathway of NO production, which enhances LPS-induced NO production^20^. We therefore cloned badger IFNγ (bdIFNγ) and expressed it in HEK293T cells. Exposure of bdMφ to culture medium containing 50 ng/ml bdIFNγ led to upregulation of TNFα mRNA but not iNOS mRNA (Fig. 1c), or release of NO. Similarly, we did not detect NO after exposing bdMφ to a mixture of LPS and bdIFNγ, or to supernatants from Concanavalin A-stimulated badger peripheral blood lymphocytes (in which upregulation of bdIFNγ mRNA was detected by QRTPCR). Furthermore, Ficoll-purified leucocytes (a mixed population of lymphocytes, monocytes and other cells) did not produce NO or upregulate iNOS mRNA after stimulation with Concanavalin A for 48 hours.

### Badgers have an intact iNOS gene but express an unusual mRNA isoform at low levels

**Figure 2:**
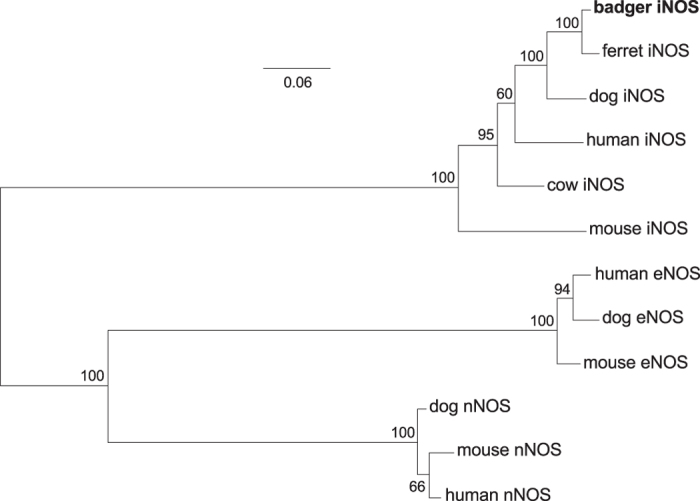
The cloned badger NOS transcript is an iNOS orthologue. Translated iNOS (NOS2) sequences were aligned to eNOS (NOS3) and nNOS (NOS1) sequences, manually trimmed to remove poorly aligned ends, and used to build a maximum likelihood tree. Branch labels indicate bootstrap support (out of 100 bootstrap replications).

Using a combination of RTPCR, 5′ RACE and genomic sequencing, we identified an in-frame coding sequence for an iNOS isoform with high homology to iNOS transcripts in other species (Fig. 2). Interestingly, the 5′ end of the transcript (corresponding to the first exon) was not homologous with the isoform generally considered the canonical 5′ iNOS sequence, but has high homology to a variant identified in RNA from human, mouse, dog and cow. Genomic sequencing revealed the potential for a transcript homologous to the canonical iNOS sequence, but this did not amplify using RTPCR or 5′ RACE. The predicted protein sequence of the observed transcript was highly conserved to rodent and human iNOS in structurally important areas and in the active site^21^ (Supplementary Fig. 2).

### Badger macrophages upregulate cytokine mRNA in response to TLR agonists and bacterial lysates

**Figure 3:**
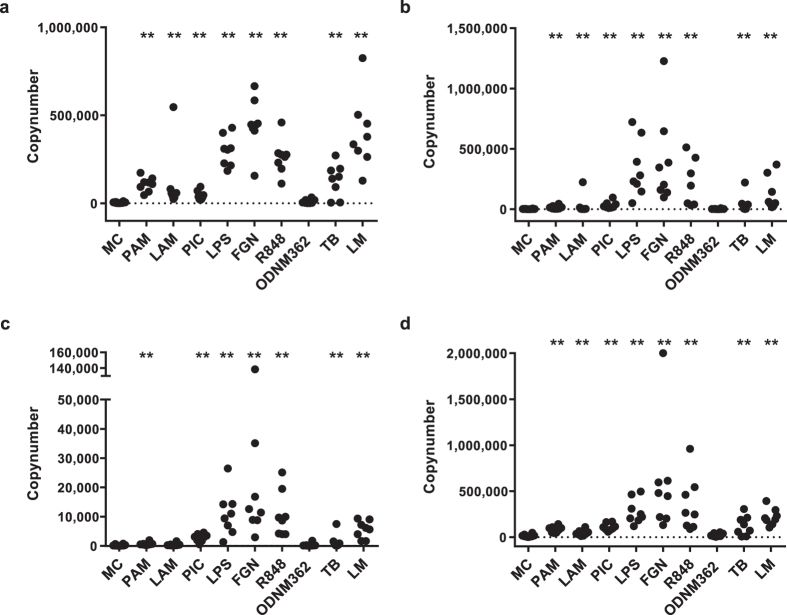
Production of cytokines by badger macrophages in response to TLR agonists. QRT-PCR was used to measure cytokine RNA in badger macrophages following TLR agonist treatment (**a**): IL1β, (**b**): IL6, (**c**): IL12, (**d**): TNFα). QRT-PCR results are given as copy number/well. GAPDH was detected at a mean of 1.1 × 10^6^ copies/well. Difference from media control (MC): *p < 0.05, **p < 0.01.

To exclude the possibility that bdMφ were simply failing to respond to TLR stimulation we examined the mRNA levels of various cytokine-encoding genes. Using the cross-species homology-based approach we cloned badger genes and developed badger-specific QRTPCR assays for GAPDH, β-actin, IL1β, TNFα, IL6, IFNγ and IL12 (Supplementary Tables 1 and 2), employing dilutions of plasmids containing the relevant gene to calculate copy numbers. Agonist-driven cytokine mRNA (IL1β, TNFα, IL6 and IL12) upregulation was evident with a wide range of TLR agonists, as with Mφ derived from other vertebrates^13^. For example, treatment of bdMφ with the TLR4 agonist LPS led to large increases in target gene copy number (mean ± Standard Error for IL1β: 292635 ± 30286; TNFα: 261197 ± 46767; IL6: 331286 ± 83210; IL12: 10827 ± 2762; Fig. 3a–d). The strongest responses occurred with LPS, FGN, R848 (agonists recognised by TLR4, 5 and 7 and/or 8) and heat killed LM. The responses generated to TLR2 agonists, PAM (a synthetic triacylated lipopeptide) and LAM were readily detectable (mean increase in IL1β copy number 102882 ± 13932 and 109799 ± 61799 respectively, although lower than for LPS (292635 ± 30286)), despite using agonist concentrations that induce comparable IL1β responses in other species^22^,^23^. Responses to a heat-killed TB preparation were similar to TLR2 agonists. The TLR3 agonist, PIC, caused low but statistically significant (p < 0.01) increases in TNFα, IL6 and IL12 mRNA. BdMφ did not respond to the TLR9 agonist ODN M362 (a synthetic unmethylated CpG) at 5 μg/ml, which stimulates responses in many vertebrates including humans^24^. Exposure of bdMφ to heat-killed *M. bovis* BCG induced upregulation of IL1β mRNA to levels comparable with those induced by exposure to heat-killed TB (Supplementary Fig. 3).

### Badger macrophages express low levels of TLR9 and weakly upregulate cytokine mRNA in response to TLR9 agonists

**Figure 4:**
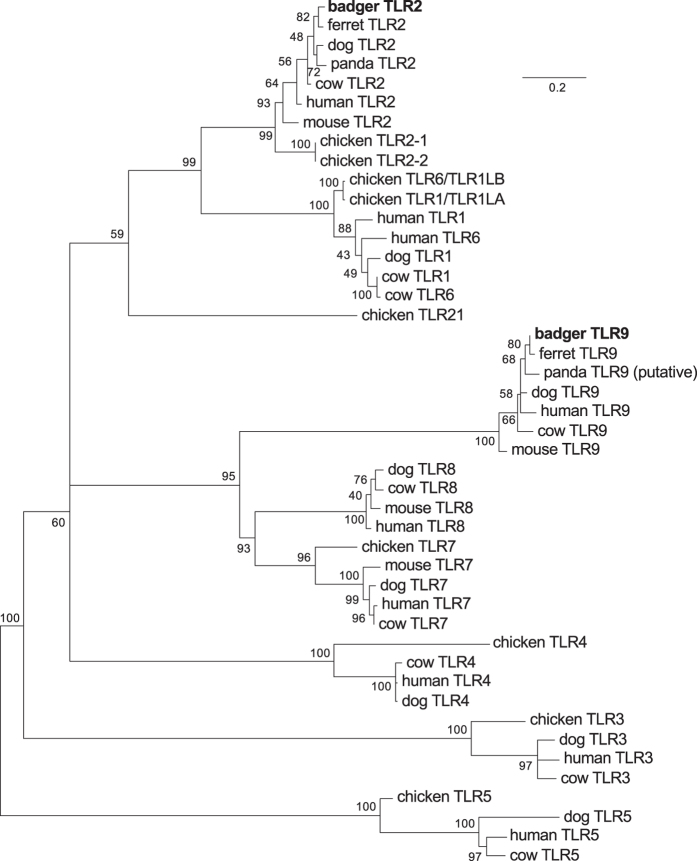
The cloned badger TLR2 and TLR9 transcripts cluster with their orthologues from other species. A peptide alignment of badger TLR2 and TLR9 TIR domains with the TIR domains of TLRs from other species was used to build a maximum likelihood tree. Branch labels indicate bootstrap support (out of 100 bootstrap replications).

**Figure 5:**
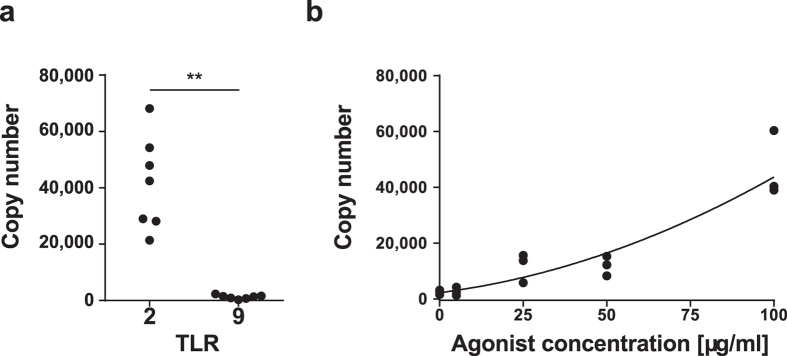
Badger macrophage TLR9 expression and response. (**a**) QRT-PCR was used to measure resting TLR RNA levels in bdMφ. GAPDH was detected at a mean of 3.3 × 10^5^ copies/well. (**b**) Production of IL1β RNA by badger macrophages stimulated with *E. coli* DNA (effect of *E.coli*, p < 0.01, fit line from linear model). GAPDH = 3.1 × 10^5^ copies/well. **p < 0.01.

Since TLR9 recognition is important during TB infections^18^ we considered it important to explore why bdMφ failed to respond effectively to ODN M362. We first sequenced badger TLR9 (and TLR2 for comparison). The sequences obtained for bdTLR9 and bdTLR2 were clear orthologues of these TLR in other species and represented complete open reading frames for both molecules (Fig. 4 and Supplementary Table 1). Secondly, we compared the level of TLR2 and TLR9 mRNA in bdMφ by QRTPCR (Fig. 5a). Notably the levels of TLR9 mRNA were much lower than detected for TLR2. Thirdly, we exposed bdMφ to a dose titration of *E. coli* DNA (5–100 μg/ml) which induced a dose dependent upregulation of IL1β (Fig. 5b) although the amounts of DNA required to elicit a response were much higher than required for induction of TLR9 dependent responses in rodents and humans^24^,^25^. NO production was not detected after stimulation of bdMφ with any dose of *E. coli* DNA.

## Discussion

Animals can adopt different strategies to resist the effects of pathogens^26^,^27^. For example, an animal might mount very strong immune responses to kill the pathogen but then suffer the effects of immune-mediated pathology. Alternatively, an animal might mount a less potent anti-pathogen immune response and tolerate the continued presence of the pathogen but avoid excessive immunopathology. One example of this strategy might involve modulation of TLR-induced responses which are critical for induction of strong immune responses but can also be responsible for life threatening immune pathology (e.g. endotoxic shock). Herein we examined the TLR response profiles of Mφ derived from wild European badgers. Badgers represent one of the most intensively studied wild mammals and are subject to considerable scrutiny in relation to their potential role in bovine tuberculosis.

Exposure of bdMφ to a wide range of TLR agonists led to upregulation of cytokine mRNA (including IL1β, IL6, IL12 and TNFα) in a similar manner to that seen with other mammals. However, TLR activation and/or exposure to IFNγ did not result in the induction of nitric oxide (NO) or an effective upregulation of iNOS mRNA. Production of NO is a feature of the immune response of many vertebrates following treatment with TLR agonists alone or in combination with cytokines, including IFNγ^28^,^29^,^30^,^31^,^32^,^33^,^34^. Human monocyte-derived macrophages have been studied intensively with different results reported^35^. Often, low levels of NO production were reported which have been attributed to peculiarities in human iNOS regulation (reviewed in ref. 36), including the lack of the LPS/IFNγ-responsive enhancer sequence present in the rodent iNOS promoter^37^, or to the methylation status of promoter elements^38^. Whether similar factors underpin the lack of effective iNOS upregulation or NO production in badgers deserves attention in future studies. A similar lack of NO production was also evident with ferret blood-derived or splenic Mφ which suggests that this NO-negative phenotype may be common amongst mustelids. High levels of NO can be harmful and the lack of an NO response might reflect a “tolerance to infection” strategy adopted by mustelids. However, it is important to note that the phenotype is targeted towards NO production since exposure of bdMφ to TLR agonists or bdIFNγ led to substantial upregulation of mRNA encoding a variety of cytokines, including TNFα (which can also be damaging). The badger iNOS gene contains an intact coding sequence, consequently the lack of iNOS upregulation or NO production could relate to transcriptional regulation, which varies between species^38^,^39^. Importantly, NO has been implicated as the major anti-*M. bovis* (bTB) killing mechanism with bovine Mφ^40^ and with *M. tuberculosis* in Mφ from other mammals^41^,^42^.

Polymorphisms in the human and bovine iNOS genes have also been linked to increased rates of *M. tuberculosis* and bTB respectively suggesting that variation in the NO response is field relevant^43^,^44^,^45^. Moreover, iNOS-deficient mice are highly susceptible to TB infection, presenting with atypical granulomas that can facilitate mycobacterial reactivation, dissemination and transmission^12^,^46^. A lack of NO production would compromise badgers’ ability to resist bTB infection and contribute to the development of atypical granulomas which have a reduced capacity to limit bTB spread.

In addition to a lack of NO, we demonstrate that bdMφ express only low levels of TLR9 and exhibit low responses to TLR9 agonists. Both TLR2 and TLR9 knockout mice have enhanced susceptibility to mycobacterial infections^18^ indicating the involvement of TLR9 recognition in protective immunity to infection with mycobacteria. TLR9 is also involved in the recognition of the anti-TB vaccine, BCG, by murine dendritic cells (DC)^47^, indeed the first stimulatory CpG motifs were isolated from BCG^48^. TLR9 mediated responses have been shown to play a role at multiple levels during TB infections including regulating granuloma formation, production of early protective type I IFN and promoting DC cross-presentation of exogenous antigen via the endogenous MHC class I pathway^49^,^50^,^51^. The very low level of TLR9 in bdMφ limits their capacity to respond to unmethylated CpG agonists and may contribute to the susceptibility of badgers to bTB, although many other factors are likely to affect bTB-badger interactions. It is important that future studies consider whether the low TLR9 phenotype we report for blood monocyte derived bdMφ is evident in other badger immune cell populations such as DC or other Mφ populations. In terms of vaccination with BCG it is noteworthy that the effective dose of BCG in badgers is 10 times larger than the human dose^10^ and this may be due to reduced recognition via TLR9. It may be possible to enhance the efficacy of BCG in badgers by supplementation with other TLR agonists. Interestingly, whereas TLR9 mediated signals enhanced DC cross-presentation, TLR2-mediated signals reduced cross-presentation and this could reduce the magnitude of protective CD8+ T cell responses^51^. Hence, the balance of TLR-mediated recognition events can be critical in the outcome of vaccination and enhanced vaccine efficacy may be achieved by employing alternative adjuvant supplements that increase cross-presentation. T cell responses can be detected after vaccination of badgers with BCG^52^ and future studies may explore the impact of alternative vaccine formulations on the magnitude and longevity of the protective CD8+ T cell response.

It is important to reiterate that bdMφ upregulate a full range of cytokines via TLR-dependent pathways, including TNFα, IL12 and IL1β^53^,^54^,^55^, which contribute to protective immunity during infection, or immunisation with BCG. Moreover, vaccination of badgers with BCG is effective against bTB under laboratory and field conditions^9^,^10^.

Our study makes two important contributions; firstly, it identifies molecular mechanisms that are likely to contribute to the susceptibility of badgers to infection with bTB: specifically, no NO and low TLR9-responses. Secondly, our study identifies TLR pathways that could be exploited to improve the design of adjuvants to enhance vaccine efficacy in badgers (e.g. supplementing with non-TLR9 agonists). In the absence of a suitable vaccine for cattle, vaccination of badgers represents a proven, sustainable and humane approach to the control of this disease in the field. Our study also highlights the importance and potential benefits of studying immune function in wildlife species threatened by zoonotic disease.

## Methods

### *Ex vivo* sampling

Badgers were blood sampled under sedation with ketamine hydrochloride (Zoetis) as part of an on-going study of their socio-ecology at Wytham Woods, Oxford, United Kingdom^56^. All badgers were released at their site of capture after sampling and full recovery. Ferrets were obtained from a recognised supplier and housed in accredited facilities before termination by Schedule 1 methods; all samples were obtained post mortem. All sampling was performed under Animals (Scientific Procedures) Act, 1986 licence and in accordance with guidance from the University of Oxford’s Animal Welfare and Ethical Review Board.

### Cell isolation and culture

Blood samples were collected into lithium heparin vacutainers (BD Biosciences) and centrifuged (1500 g, 10 min, 4 °C) to isolate plasma. Peripheral blood mononuclear cells were isolated following established procedures^57^. Briefly, cells were isolated using Ficoll (Life Technologies), washed and resuspended in culture medium comprising phenol red-free Dulbecco’s Modified Eagle Medium (Life Technologies) containing 10% FCS (Foetal Calf Serum, Life Technologies), 2 mM Penicillin and Streptomycin (Life Technologies), and 2 mM L-Glutamine (Sigma-Aldrich). Cells were plated at 5 × 10^5^ cells/well on 96 well plates and incubated for 24 hours prior to removal of non-adherent cells by washing with medium. Following a further 24 hours incubation cells were either mock-treated or treated with defined TLR agonists, microbial lysates (all obtained from Invivogen, concentrations given in Supplementary Table (3) or recombinant IFNγ (50 ng/ml). For NO assays and IFNγ bioactivity assays, cells were cultured under treatment conditions as described for 48 hours; medium was removed and frozen for Griess assay (Promega). For TLR agonist induced cytokine analyses, cells were stimulated with agonist for 4 hours, following initial time course studies (Supplementary Fig. 4). Points shown in figures represent replicate wells where cell lines are analysed (Fig. 1a, HD11 and RAW), and cells derived from individual badgers in all other cases.

### RNA extraction and cDNA synthesis

RNA was extracted from cells frozen in RLT buffer (Qiagen) using either single columns or 96 well format columns (both Qiagen) following the manufacturer’s spin protocol. For QRT-PCR, the input RNA amount was normalised for cDNA reactions across a sample set and cDNA was generated (Life Techologies).

### Badger gene sequences

Primers were designed based upon regions of target genes conserved in multiple mammalian genomes. Appropriate primer sets were used to amplify fragments of badger cytokine or TLR genes from PBMC cDNA using Myfi PCR mix (Bioline), according to manufacturer’s instructions. Products were cloned into pGEM TEasy (Promega) and sequenced using plasmid targeting (M13) or gene specific primers with BigDye chemistry (Life Technologies), according to manufacturer’s instructions. Sequences were analysed using Bioedit^58^, and badger specific sequence used to design primers for 5′RACE PCR. 5′RACE cDNA was generated using a SMARTer RACE kit (Clontech) according to manufacturer’s instructions. PCR products generated from RACE PCR products were cloned into pGEM TEasy, as described, or into pTarget (Promega) if longer than 1 kb, and sequenced as above (European Nucleotide Archive accession numbers are given in Supplementary Table 1). Sequences were analysed using BioEdit and MEGA^59^ and compared to human, mouse dog and ferret sequences to identify target regions for intron spanning QRT-PCR assays.

### QRT-PCR assays

Intron spanning SYBR green QRT-PCR assays were designed using badger gene sequences (Supplementary Table 2). Assays were tested for specificity by melt curve analysis, and cloning and sequencing of amplicons. Validated assays were then used to analyse cytokine cDNA levels in agonist treated PBMC. Dilution series of linearised plasmids containing cytokine or TLR gene sequences were used to calculate copy numbers. Ct values for TLR or cytokine target amplification for each sample were adjusted using the GAPDH Ct value for the same sample, to account for variation in sampling and RNA preparation. The slopes of a plot of Ct against log_10_ of the plasmid dilution series present on each plate were calculated. The slopes of the respective gene of interest (GOI) and GAPDH dilution series were used to calculate GOI Ct values and adjusted for differences in input total RNA as follows: corrected Ct value = Ct + (Nt−Ct’) 3 S/S’, where Ct is the GOI Ct value, Nt is the median GAPDH Ct for all samples within an experiment, Ct’ is the GAPDH value of the individual sample, S is the GOI slope, and S’ is the GAPDH slope^60^. A 10-fold dilution series of linearised plasmid DNA was prepared for each target, starting at 20 million copies. Ct values for the dilution series were calculated as described for samples. Plots of 40 – Ct against copy number of the serial dilution were used to derive sample copy numbers per well using the following equation: sample copy number = y*e^(ab)^, where “y” is the y intercept, “a” represents the slope of the dilution series and “b” is the corrected 40-Ct of the sample.

### Recombinant IFNγ

The published sequence^61^ for full-length badger IFNγ including Kozak and signal sequences was modified to encode 2 glycine linker residues and a 6 histidine residue tag at the C terminus, and synthesised (Geneart). The sequence was subcloned into pTarget (Invivogen) using standard molecular biology techniques, and HEK293T cells were transiently transfected using TransIT-2020 (Mirus Bio) and cultured in Freestyle protein-free medium (ThermoFisher Scientific). The presence of a protein of the expected molecular weight (~18 kDa) in cell culture supernatants was confirmed by Western Blot, using Penta-His antibody (Qiagen), and quantified using a dot blot with the same reagent, by comparison with a polyhistidine-tagged protein of known concentration. It was used to treat cells at 50 ng/ml.

### Statistics

Statistical analyses were performed in R 3.1.2^62^. Responses to agonist panels (Figs 1b and 3) were analysed using linear mixed effects models with individual badger as a random effect, and the dose response titration of *E. coli* DNA (Fig. 5b) was analysed using a linear model, because a random effect on individual badger was not significant. Where necessary, data were transformed in order to meet assumptions of normality and homoscedasticity of residuals. Probabilities were calculated from linear mixed effects models using the Satterthwaite approximation for degrees of freedom^63^, as implemented in the lmerTest package^64^. Where more than one measurement was involved (Figs 1b and 3), a Holm–Bonferroni^65^ family-wise error rate correction was applied using function p.adjust(). Mixed models were built using the using the lme4 package^66^, and the linear model using function l m(). TLR2 and TLR9 expression levels (Fig. 5a) were compared using a two-tailed paired t test. Nitric oxide production data (Fig. 1a) was compared using a two-tailed paired t test (function t.test()) with a Holm–Bonferroni correction. IFNγ response (Fig. 1c) data could not be conformed to assumptions for parametric tests, so distributions were compared using one-tailed Kolmogorov–Smirnov tests^67^, with a Bonferroni correction for multiple comparisons.

### Sequence alignments

NOS sequences were aligned using Clustal Omega^68^ and manually refined, with trimming to remove unaligned or poorly aligned stretches at the N and C termini. The sub-alignment of iNOS sequences was formatted for publication using BioEdit 7.2^58^ and CorelDRAW X5. Badger iNOS transcript as described in the main text. Other sequence data was drawn from publically available databases (see Supplementary methods): RefSeq Release 72^69^; Genbank^70^; Ensembl release 82^71^ Dog genome assembly CanFam3.1 and Ferret genome assembly MusPutFur1.0. Mouse and human iNOS transcripts proved the forms most closely corresponding to the badger transcript which are present in publically available databases. Each is supported by RNASeq/EST evidence. TIR domains were identified in TLR peptide sequences using SMART^72^, and aligned using Clustal Omega. TIR domains were aligned in preference to whole-sequence alignments as functional constraints mean that TIR domains can be aligned more reliably than other regions of TLRs^73^,^74^.

### Phylogenetic trees

MEGA 6.06^59^ was used for model selection and maximum likelihood tree construction, with 100 bootstrap replications. For the NOS tree (Fig. 2), a JTT + G model (Jones-Taylor-Thornton, with rates among sites gamma distributed) was employed. For the TIR domain tree (Fig. 4), an LG + G + I model (Le and Gascuel, with rates among sites gamma distributed, and with invariant sites) was used. In both cases, five discrete gamma categories were used, gaps were partially deleted (95% coverage cutoff), and a moderate branch swap filter was used. Default tree inference options were used. Altering the number of gamma categories or strength branch swap filter, or completely deleting sites with gaps, did not affect the branching of the trees.

## Additional Information

**How to cite this article**: Bilham, K. *et al*. Badger macrophages fail to produce nitric oxide, a key anti-mycobacterial effector molecule. *Sci. Rep.* **7**, 45470; doi: 10.1038/srep45470 (2017).

**Publisher's note:** Springer Nature remains neutral with regard to jurisdictional claims in published maps and institutional affiliations.

## Supplementary Material

Supplementary Information
