# Supplementary material for: Badger macrophages fail to produce nitric oxide, a key anti-mycobacterial effector molecule
Source: Sci Rep. 2017 Apr 6;7:45470. doi: 10.1038/srep45470 (PMC5382539; doi:10.1038/srep45470)
Supplement: Supplementary Information [file srep45470-s1.pdf]

Supplementary Information

Title: Badger macrophages fail to produce nitric oxide, a key anti-mycobacterial effector molecule.

Authors: Kirstin Bilham[‡], Amy C. Boyd[‡], Stephen G. Preston, Christina D. Buesching, Chris Newman, David W. Macdonald and Adrian L. Smith.

[‡]These authors contributed equally to this work.

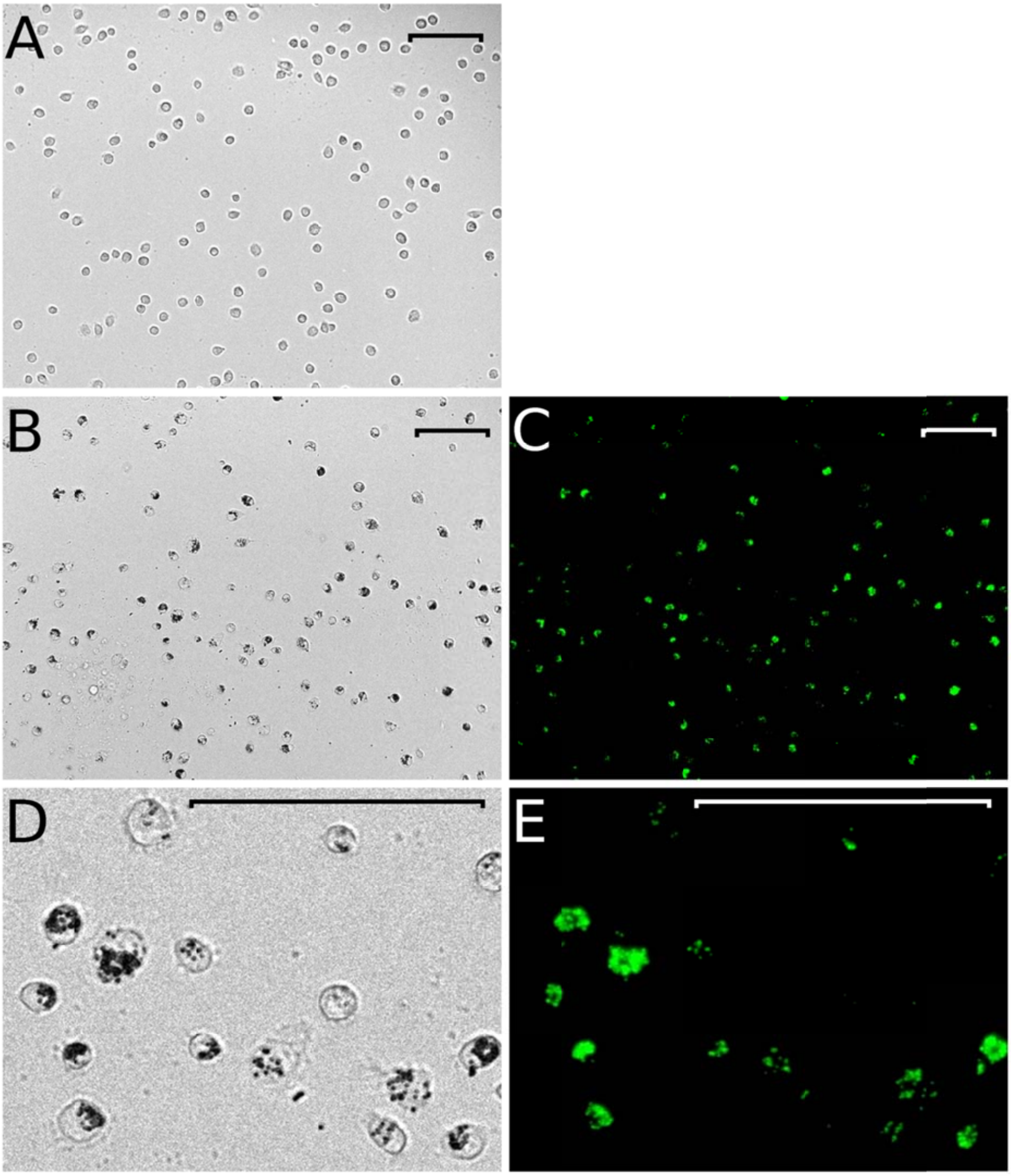

Supplementary Figure 1. Cultured badger peripheral blood monocyte-derived adherent cells are highly phagocytic. Cells cultured for 48 hours at 37^{0}C 5% CO_2 without beads (a) or for the final 4 hours with fluorescent latex beads (b, c, d e). Bright field (a, b and d) and fluorescent micrographs (c and e) are depicted. Images a, b, and c at low magnification with d and e depicting a sub-section of b and c at higher magnification. Scale bars in each panel represent 100 μm.

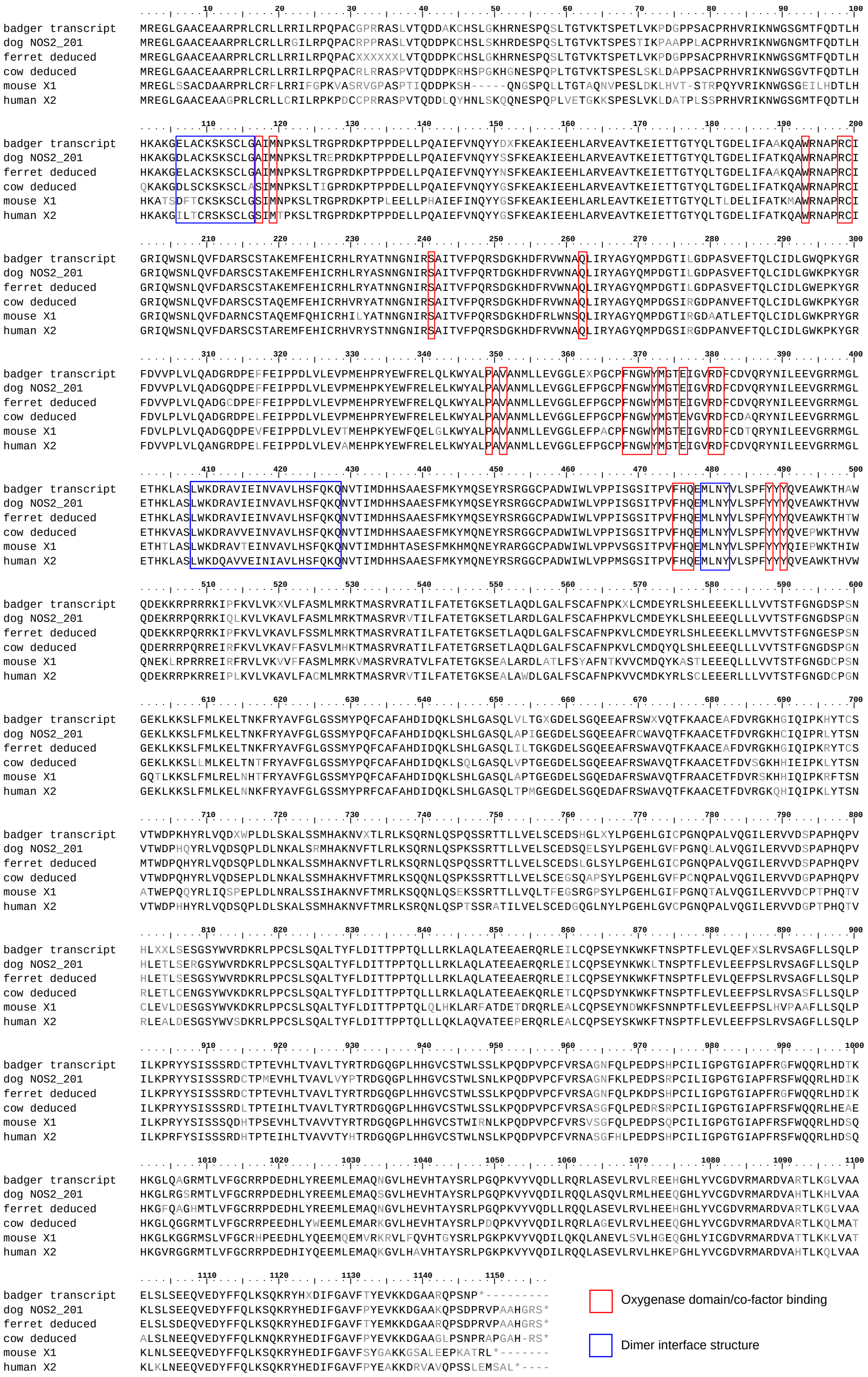

Supplementary Figure 2. Translation of the badger iNOS nucleotide sequence shows that residues contributing to dimer formation and catalytic activity are well-conserved. The identified badger transcript was translated and aligned to corresponding transcripts, or hypothetical transcripts, from other species. Coloured boxes show sites reported to be involved in dimer formation (blue) or in the oxygenase domain/co-factor binding (red). Residues in black are well conserved (BLOSUM62 score ≥1).

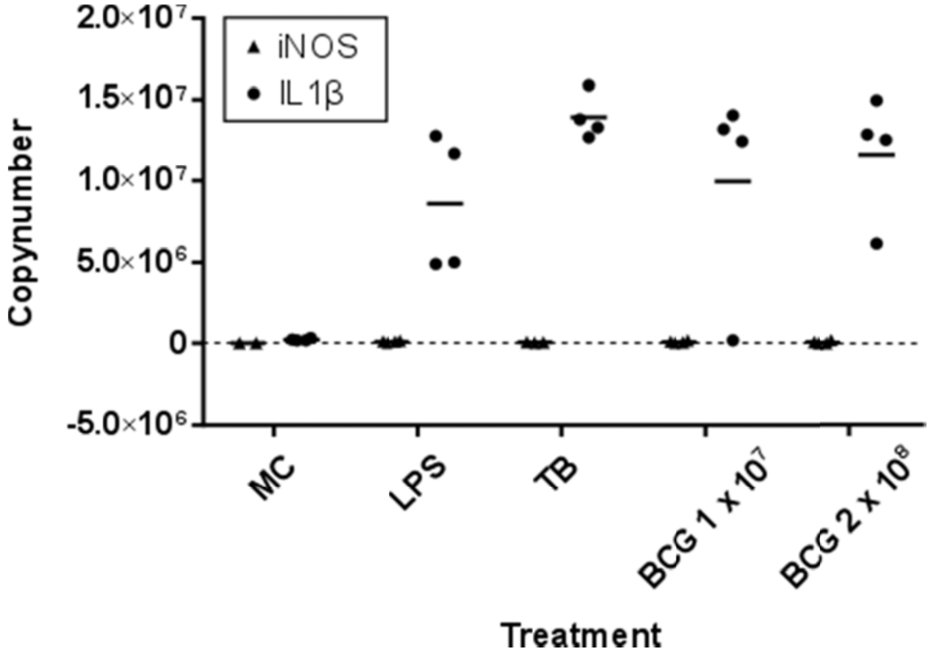

Supplementary Figure 3. Production of cytokines by badger macrophages in response to LPS, TB lysate and heat killed BCG. Cells were cultured for 48 hours prior to stimulation for 4 hours. MC= media control, LPS= lipopolysaccharide at 1ug/ml, TB= heat killed *M. tuberculosis*, BCG= heat killed *M. bovis* Bacillus Calmette-Guerin All data displayed as copy number generated by comparison with a standard curve of the relevant target, each point represents one individual and lines indicate the mean.

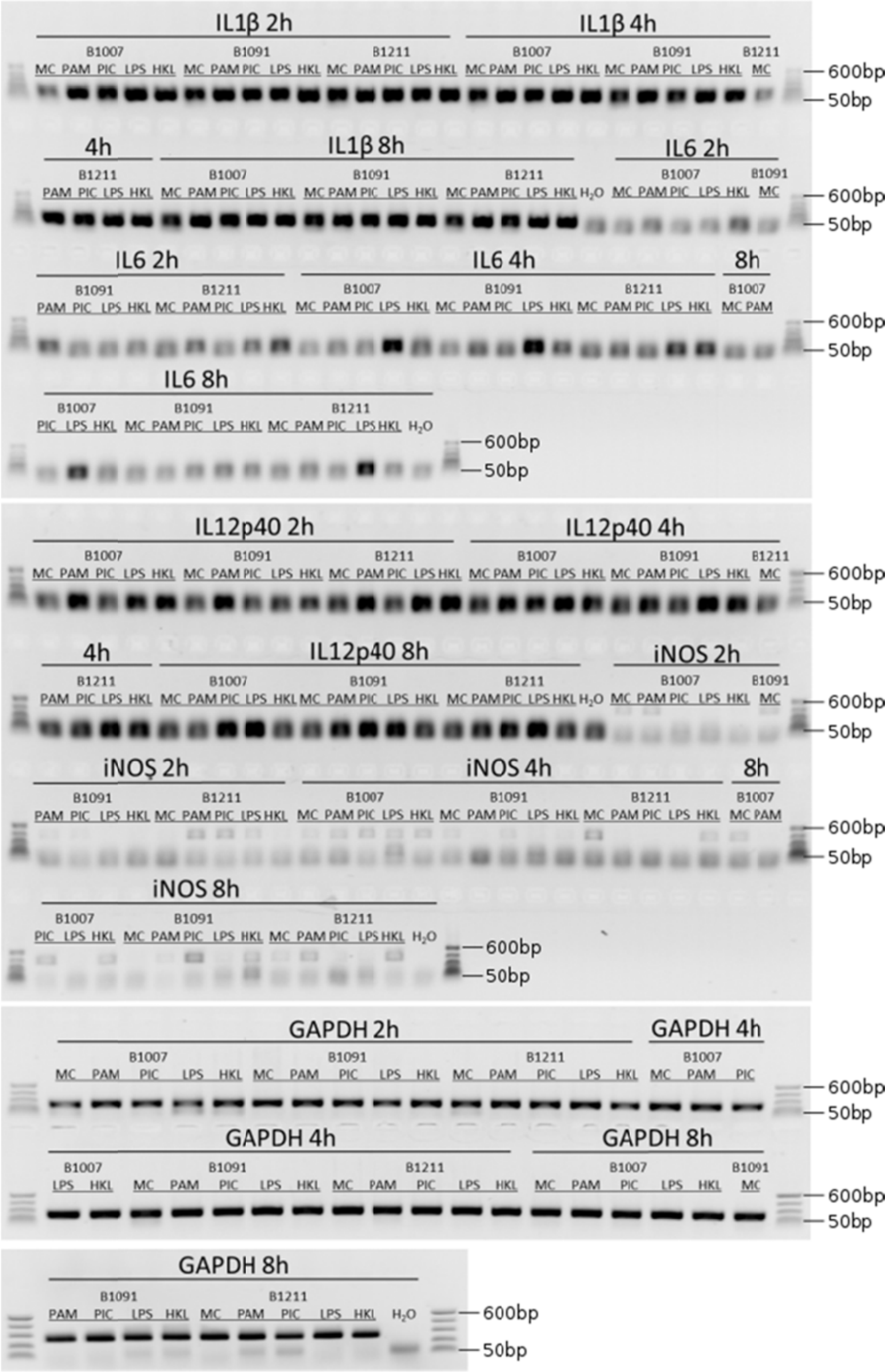

Supplementary Figure 4. Initial RTPCR time course analyses of badger innate immune genes. Cytokine mRNA (IL1, IL6 and IL12) and GAPDH mRNA levels was assayed by RT-PCR at 2, 4 and 8 hours (h) post stimulation with Pam3CSK4 (PAM), PolyI:C (PIC), lipopolysaccharide (LPS) and heat killed Listeria (HKL) at concentrations given in Supp Table 3.. MC denotes media control. B1007, B1091 and B1211 are individual badger IDs.

Supplementary Table 1 Badger gene accession numbers (submitted to the European Nucleotide Archive).

Accession number	Gene
LT821080	Interleukin 1 beta
LT821081	Interleukin 6
LT821082	Interleukin 12p40
LT821083	Toll-like receptor 2
LT821084	Toll-like receptor 9
LT821085	Interferon gamma
LT821086	Tumour necrosis factor alpha
LT821087	Glyceraldehyde 3-phosphate dehydrogenase
LT821088	Inducible nitric oxide synthase

Supplementary Table 2 QRT-PCR primer sequences

Target (F:forward, R:reverse)	Sequence 5'-3'
GAPDH_F	GGCGCTTGGTCACCAG
GAPDH_R	GCAGAAGGAGCGGAGATGATG
IL12_F	CATTTGGGAACTGGAGAAAA
IL12_ R	ACCGGAGCCTAAGACTTCAC
TNFα_F	CCTGCTGCACTTTGGAGTGATC
TNFα_R	CTATTAGCTGGTTGTCTGTTAGCTCCAC
IL6_F	GAACTCCCTCTCCACAAGCG
IL6_R	GCCAGTGCCTCCTTGCTG
IFNγ_F	GAAGAACTGGAGAGAGGAGAGTGAC
IFNγ_R	CCGCTTACTGCTGCTGCTATTG
IL1β_F	CTGTACCTGTCCTGTGTGATGAAAG
IL1β_R	GTTATGTCCTGACCACCTCTGTTATTTCC
iNOS_F	CCTGTGTTCCACCAGGAGAT
iNOS_R	CTTCTCCGGGGTCTCTTCTT
TLR2_F	CTTTTATTTCCCTGCGGAGTC
TLR2_R	TTCCCCGAGGGATTTGTAAC
TLR9_F	CAGCTGCTGCCTAGTTTGC
TLR9_R	TGCACCAGGAGAGACAAGG

Supplementary Table 3 Working concentrations of TLR agonists (Invivogen)

TLR targeted	TLR agonist	Concentration
TLR 2	Pam3CSK4	300 ng/ml
TLR 2	Lipoarabinomannan	1 µg/ml
TLR 3	Poly (I:C)	12.5 µg/ml
TLR 4	Lipopolysaccharide	1 µg/ml
TLR 5	Flagellin	10 µg/ml
TLR 7	R848/Imiquimod	1 µg/ml
TLR 9	ODN M362	5 µg/ml
TLR 9	*E. coli* DNA	50 µg/ml
Multiple	Heat killed *Mycobacterium tuberculosis*	1 µg/ml
Multiple	Heat killed *Listeria monocytogenes*	10^7-10^8 cells/ml
Multiple	Heat killed Bacillus Calmette–Guérin	10^7- 2 x 10^8 cells/ml

Supplementary Methods

Microscopy. Adherent cells were cultured as described in materials and methods. Fluorescent labelled beads (L1030; Sigma, UK) were added to culture medium at 1:1000 v/v according to manufacturer's instructions. Images were taken using a ZOE Fluorescent Cell Imager (Bio-Rad, UK) after 4 hours.

Sequences. Mouse and human iNOS transcripts are the forms most closely corresponding to the badger transcript which are present in publically available databases. Each is supported by RNASeq/EST evidence. Mouse transcript X1 = RefSeq XM_006532446; human transcript X2 = RefSeq XM_011524860. Dog NOS2_201 is Ensembl transcript ID ENSCAFT00000046006 (automatic prediction from genomic sequence). Ferret iNOS transcript was deduced by ourselves, with Emsembl genomic sequence GL896917.1: 5,007,764-5,007,959 joined to Ensembl automatically predicted transcript ENSMPUT00000014484. Cow transcript was deduced from a combination of EST data (5' end: GenBank DN741727.1) and iNOS RefSeq sequence NM_001076799.1.

eNOS and nNOS sequences are from RefSeq: mouse eNOS = NM_008713.4; human eNOS = NM_000603.4; dog eNOS = NM_001003158.2; mouse nNOS = NM_008712.3; human nNOS = NM_000620.4; dog nNOS = NM_001195145.1.

Badger TLR sequences are as described in the main text. Other TLR sequences are from RefSeq: human TLR1 = NM_003263.3; human TLR2 = NM_003264.3; human TLR3 = NM_003265.2; human TLR4 = NM_138554.4; human TLR5 = NM_003268.5; human TLR6 = NM_006068.4; human TLR7 = NM_016562.3; human TLR8 = NM_138636.5; human TLR9 = NM_017442.3; mouse TLR2 = NM_011905.3; mouse TLR7 = NM_001290757.1; mouse TLR8 = NM_133212.3; mouse TLR9 = NM_031178.2; dog TLR1 = NM_001146143.1; dog TLR2 = NM_001005264.3; dog TLR4 = NM_001002950.2; dog

TLR6 = NP_001041589.1; dog TLR7 = NP_001041589.1 ; dog TLR9 = NM_001002998.1; chicken TLR1/TLR1LA = NM_001007488.4; chicken TLR2-1 = NM_204278.1; chicken TLR2-2 = NM_001161650.1; chicken TLR3 = NM_001011691.3; chicken TLR4 = NP_001025864.1; chicken TLR5 = NP_001019757.1; chicken TLR6/TLR1LB = NP_001075178.3; chicken TLR7 = NM_001011688.2; chicken TLR21 = NM_001030558.1; cow TLR1 = NM_001046504.1; cow TLR2 = NM_174197.2; cow TLR3 = NM_001008664.1; cow TLR4 = NM_174198.6; cow TLR5 = NM_001040501.1; cow TLR6 = NM_001001159.1; cow TLR7 = NM_001033761.1; cow TLR8 = NM_001033937.1; cow TLR9 = NM_183081.1. Or from UniProt[74]: dog TLR3 = E2RIV9-1; dog TLR5 = F1Q0H5-1; dog TLR8 = F1PEB2-1; ferret TLR2 = D7F066; ferret TLR9 = G9KTJ9. Or from predicted transcripts from Ensembl release 82 (giant panda genome assembly ailMel1 / ferret genome assembly MusPutFur1.0): panda TLR2 = ENSAMET00000020274; panda TLR9 (putative, identified by BLAT search) = ENSAMET00000009973; ferret TLR2 = ENSMPUT00000019046; ferret TLR9 = ENSMPUT00000011807.
